# Enhancement of chemotherapy by manipulation of tumour pH

**DOI:** 10.1038/sj.bjc.6690455

**Published:** 1999-06

**Authors:** N Raghunand, X He, R van Sluis, B Mahoney, B Baggett, C W Taylor, G Paine-Murrieta, D Roe, Z M Bhujwalla, R J Gillies

**Affiliations:** Arizona Cancer Center, 1501 N Campbell Avenue, Tucson, AZ 85724-5024, USA; Department of Radiology, Johns Hopkins School of Medicine, Baltimore, MD, USA

**Keywords:** doxorubicin, acid-base balance, chemotherapy, MRS, bicarbonate

## Abstract

The extracellular (interstitial) pH (pHe) of solid tumours is significantly more acidic compared to normal tissues. In-vitro, low pH reduces the uptake of weakly basic chemotherapeutic drugs and, hence, reduces their cytotoxicity. This phenomenon has been postulated to contribute to a ‘physiological’ resistance to weakly basic drugs in vivo. Doxorubicin is a weak base chemotherapeutic agent that is commonly used in combination chemotherapy to clinically treat breast cancers. This report demonstrates that MCF-7 human breast cancer cells in vitro are more susceptible to doxorubicin toxicity at pH 7.4, compared to pH 6.8. Furthermore ^31^P-magnetic resonance spectroscopy (MRS) has shown that the pHe of MCF-7 human breast cancer xenografts can be effectively and significantly raised with sodium bicarbonate in drinking water. The bicarbonate-induced extracellular alkalinization leads to significant improvements in the therapeutic effectiveness of doxorubicin against MCF-7 xenografts in vivo. Although physiological resistance to weakly basic chemotherapeutics is well-documented in vitro and in theory, these data represent the first in vivo demonstration of this important phenomenon. © 1999 Cancer Research Campaign


British Journal of Cancer (1999) **80**(7), 1005–1011
© 1999 Cancer Research Campaign
Article no. bjoc.1998.0455

### Enhancement of chemotherapy by manipulation of tumour pH

**N Raghunand[1,*], X He[1,*], R van Sluis[1], B Mahoney[1], B Baggett[1], CW Taylor[1], G Paine-Murrieta[1], D Roe[1], ZM Bhujwalla[2] and RJ Gillies[1]**

[1]Arizona Cancer Center, 1501 N Campbell Avenue, Tucson, AZ 85724-5024, USA; [2]Department of Radiology, Johns Hopkins School of Medicine, Baltimore, MD, USA

**Summary** The extracellular (interstitial) pH (pHe) of solid tumours is significantly more acidic compared to normal tissues. In-vitro, low pH reduces the uptake of weakly basic chemotherapeutic drugs and, hence, reduces their cytotoxicity. This phenomenon has been postulated to contribute to a 'physiological' resistance to weakly basic drugs in vivo. Doxorubicin is a weak base chemotherapeutic agent that is commonly used in combination chemotherapy to clinically treat breast cancers. This report demonstrates that MCF-7 human breast cancer cells in vitro are more susceptible to doxorubicin toxicity at pH 7.4, compared to pH 6.8. Furthermore $^{31}$P-magnetic resonance spectroscopy (MRS) has shown that the pHe of MCF-7 human breast cancer xenografts can be effectively and significantly raised with sodium bicarbonate in drinking water. The bicarbonate-induced extracellular alkalinization leads to significant improvements in the therapeutic effectiveness of doxorubicin against MCF-7 xenografts in vivo. Although physiological resistance to weakly basic chemotherapeutics is well-documented in vitro and in theory, these data represent the first in vivo demonstration of this important phenomenon.

**Keywords:** doxorubicin; acid-base balance; chemotherapy; MRS; bicarbonate

In the early part of this century, Otto Warburg postulated that tumours were acidic because the rate at which they produced lactic acid was 'remarkable' (Warburg, 1956). Subsequent measurement of pH in a variety of solid tumours with microelectrodes apparently confirmed this hypothesis (Wike-Hooley et al, 1984). It is assumed that microelectrode measurements primarily interrogate extracellular (interstitial) volumes. However, in-vivo $^{31}$P magnetic resonance spectroscopy (MRS) of endogenous intracellular inorganic phosphate (Pi) measures an intracellular pH (pHi) which is neutral-to-alkaline in the same or similar tumours (Griffiths, 1991; Negendank, 1992). Thus, the apparent acidity of the extracellular (interstitial) pH (pHe) does not translate to an acidic pHi. Recently, $^{31}$P MRS methods have been developed to simultaneously measure the pHi and pHe of tumours using endogenous Pi and exogenous 3-aminopropylphosphonate (3-APP), respectively (Gillies et al, 1994). This method has clearly shown, in a number of solid tumour xenografts, that the pHi of tumour cells is neutral–alkaline, while the pHe of the same tumours is acidic (McCoy et al, 1995; Raghunand et al, 1999a). Thus, solid tumours generally have substantial acid-outside pH gradients.

Acid-outside pH gradients have a number of important sequellae that are germane to cancer, including a theoretically reduced partitioning of weakly basic chemotherapeutic drugs into the relatively alkaline cells. This phenomenon has its basis in the 'ion-trapping' hypothesis wherein weak bases partition into, and are sequestered by, acidic compartments, such as the extracellular fluid (Roos, 1978). This occurs because uncharged, organic free bases are much more permeable than their protonated and charged counterparts and establish equal concentrations on both sides of the membrane (Gillies and Deamer, 1978). Since the ratio of charged–uncharged species increases with lower pH, more total base is found in acidic compartments. This hypothesis is applicable to weakly basic chemotherapeutic drugs, such as doxorubicin (pKa = 7.6), which is one of the most widely prescribed antineoplastic agents used in the treatment of breast cancer (Taylor et al, 1991).

*Received 24 August 1998*
*Revised 3 December 1998*
*Accepted 7 December 1998*

*Correspondence to*: RJ Gillies

### MATERIALS AND METHODS

### Cell and tumour growth

MCF-7 cells were obtained from W S Dalton (Arizona Cancer Center) and cultured in RPMI-1640/DMEM (Dulbecco's modified Eagle's medium) supplemented with 10% fetal bovine serum (FBS) (HyClone, Logan, UT, USA). For in vivo culturing, a suspension of $5 \times 10^6$ MCF-7 cells in 0.05 ml of Hank's balanced salt solution were implanted in the mammary fat pads of 6- to 7-week-old female severe combined immunodeficient (SCID) mice. Since MCF-7 cells are oestrogen-dependent, 17β-oestradiol pellets (0.72 mg, 60-day release; Innovative Research of America, Sarasota, FL, USA) were subcutaneously implanted in the shoulder region of the mice by means of a 12-gauge trochar (Innovative Research) 2 days prior to tumour inoculation.

### Magnetic resonance spectroscopy

Prior to MRS, mice were anaesthetized with a combination of ketamine (72 mg $kg^{-1}$), xylazine (6 mg $kg^{-1}$) and acepromazine (6 mg $kg^{-1}$). A 3/4″, 24-gauge catheter (Elf Sanofi Inc., Overland Park, KS, USA), connected to a 1.58 mm intradermal (i.d.)

*These authors contributed equally to this work.

polyethylene tube (Becton Dickinson, Parsippany, NJ, USA), long enough to extend out of the magnet was inserted into the intraperitoneal (i.p.) cavity of the anaesthetized animal. The mouse was then immobilized on a home-built probe with a coil tunable to $^{1}H$ or $^{31}P$. After shimming the magnet, 3-APP (0.15–0.3 ml, 128 mg $ml^{-1}$, pH 7.4) was injected into the mouse via the i.p. catheter. Volume-selective $^{31}P$ spectra were acquired at 4.7 T on a Bruker Biospec with the ISIS sequence (Ordidge et al, 1988) with adiabatic slice-selective and excitation pulses repeated every 10–12 s, using a gradient strength of 75 mT $m^{-1}$. In all cases, a dwell time of 62.5 μs was employed, and 8192 data points were collected from 184 transients. Scout images of the tumour and surrounding abdominal tissue were obtained each time in order to guide the positioning of the voxel. The large spectral widths employed resulted in up to a 1 mm difference in the positioning of the voxel containing either α-NTP or 3-APP, and the voxel containing the central frequency. This chemical shift artefact was not corrected for, but voxel sizes and placement were chosen so as to minimize the contribution of signal arising from the mouse body wall, while covering as much of the tumour as possible.

### Measurement of intracellular pH in vitro

Intracellular pH (pHi) was measured in vitro using the fluorescent dye, SNARF-1, as described previously (Martinez-Zaguilan et al, 1991). Briefly, MCF-7 cells were grown onto 9 × 22 mm glass coverslips, washed three times with buffer A (1.3 mM calcium chloride, 1 mM magnesium sulphate, 5.4 mM potassium chloride, 0.44 mM $KH_2PO_4$, 110 mM sodium chloride, 0.35 mM $NaH_2PO_4$, 5 mM glucose, 2 mM glutamine and 5 mM HEPES, 5 mM MES, 10 mM sodium hydrogen carbonate ($NaHCO_3$) at a pH of 7.15 at 37°C and subsequently incubated for 30 min at 37°C, in a 5% carbon dioxide atmosphere with 3 ml of buffer A containing 10 μM acetoxymethylester SNARF-1 (Molecular Probes, Eugene, OR, USA). This was followed by a second incubation in buffer A for 45 min to allow for complete hydrolysis of the dye. Coverslips were then placed in a holder/perfusion device and inserted into a fluorometer cuvette and fluorescence measurements were acquired at an excitation of 534 nm with the emission sequentially sampled at 584 and 644 nm in an SLM8000C (SLM, Urbana, IL, USA). The ratio (R) of fluorescence intensities of emissions at 584 and 644 nm was converted to pHi values using the equation: $pH = 7.38 + \log_{10} 0.822 + \log_{10} [(R - 0.458)/(1.928 - R)]$. Data are presented as mean ± s.e.m. of six independent measurements.

### Doxorubicin uptake and cytotoxicity in vitro

MCF-7 cells were grown to confluence in 6-well plates at which time the cells were incubated in DMEM/F12 containing 20 mM HEPES, 20 mM MES, 10% FBS and 0.208 μCi per well of $^{14}C$ doxorubicin (Amersham) for 30 min at 37°C in a 5% carbon dioxide atmosphere at pH of 6.8 and 7.4 respectively. After the incubation, the plates were placed on ice and washed five times with ice-cold HBSS, followed by extraction with 1.0 ml of 0.1 N NaOH for 1 h at 4°C. Samples were divided into equal aliquots for determination of protein content using the Bradford assay (Bio-Rad), and radioactivity using liquid scintillation counting.

Cytotoxicity was determined as previously described (Gillies et al, 1986). Briefly, cells were grown to log phase in 96-well plates and medium was exchanged for one at either pH 6.8 or 7.4 containing the indicated concentrations of doxorubicin. Medium pH was buffered using non-volatile buffers (10 mM MES, 20 mM HEPES and 10 mM TRICINE) in combination with bicarbonate concentrations that were adjusted to be in equilibrium with 5% ambient carbon dioxide. Twenty-four hours later, regular growth medium was replaced and cells allowed to grow a subsequent 72 h, after which time they were fixed and stained with crystal violet for determination of cell number.

**Table 1** Theoretical and measured enhancement of doxorubicin uptake caused by increasing extracellular pH in vitro and in vivo

| In vitro | Low pHe | High pHe |
|---|---|---|
| Extracellular pH | 6.8 | 7.4 |
| Intracellular pH (*n*) | 7.05 ± 0.06 (22) | 7.20 ± 0.07 (5) |
| Theoretical ratio | 0.69 ± 0.07 | 1.36 ± 0.17 |
| Theoretical enhancement | | 1.97 ± 0.45 |
| Doxorubicin uptake (*n*) | 65.1 ± 2.7 (6) | 166.9 ± 9.1 (6) |
| Uptake enhancement | | 2.56 ± 0.25 |
| $EC_{50}$ (μM) | 0.27 | 0.12 |
| Toxicity enhancement | | 2.25 |
| **In vivo** | **Control** | **+ $NaHCO_3$** |
| pHe (800 $mm^3$ tumour) | 6.99 ± 0.11 | 7.84 ± 0.13 |
| pHi (800 $mm^3$ tumour) | 7.15 ± 0.08 | 7.39 ± 0.13 |
| pH gradient | +0.16 | –0.45 |
| Theoretical ratio | 0.6 | 1.6 |
| Theoretical enhancement | | 2.5 |

**Table 2** Tumour growth statistics

| | A | B | C | D |
|---|---|---|---|---|
| No. mice | 17 | 13 | 12 | 13 |
| T–C (d to 300 $mm^3$) | – | – | 15 | 22 |
| T/C (%) | – | – | 0.47 | 0.29 |
| Growth rate ($d^{-1}$) | 0.206 | 0.215 | 0.156 | 0.092 |
| Standard deviation | 0.094 | 0.060 | 0.067 | 0.047 |
| *P* vs group A | – | 0.732 | 0.031 | 0.002 |
| *P* vs group B | 0.732 | – | 0.027 | 0.001 |
| *P* vs group C | 0.031 | 0.027 | – | 0.008 |
| *P* vs group D | 0.002 | 0.001 | 0.008 | – |

### Calculation of theoretical drug distributions

Cytoplasmic–extracellular drug ratios were calculated at the steady-state using methods described previously (Roos, 1978; Raghunand et al, 1999*b*). Briefly, the ratio of protonated (charged) to deprotonated (uncharged) doxorubicin were calculated from the Henderson–Hasselbach equation in both intracellular and extracellular compartments using the pHi and pHe, respectively, and a drug pKa of 7.6. The concentration of the uncharged specie was set to 1.0 on both sides of the membrane and the total concentration was calculated as the sum of (charged + uncharged). Data were expressed as a ratio of intra- to extracellular concentrations. Enhancement was calculated as the ratio at high pH, relative to that at low pH.

### Tumour growth statistics

Tumour size was measured with calipers and calculated as: $[(\text{width})^2 \times \text{length}]/2$. Data were linearized by converting volumes (in $mm^3$) to their cube roots. The least squares regression line of

 © 1999 Cancer Research Campaign

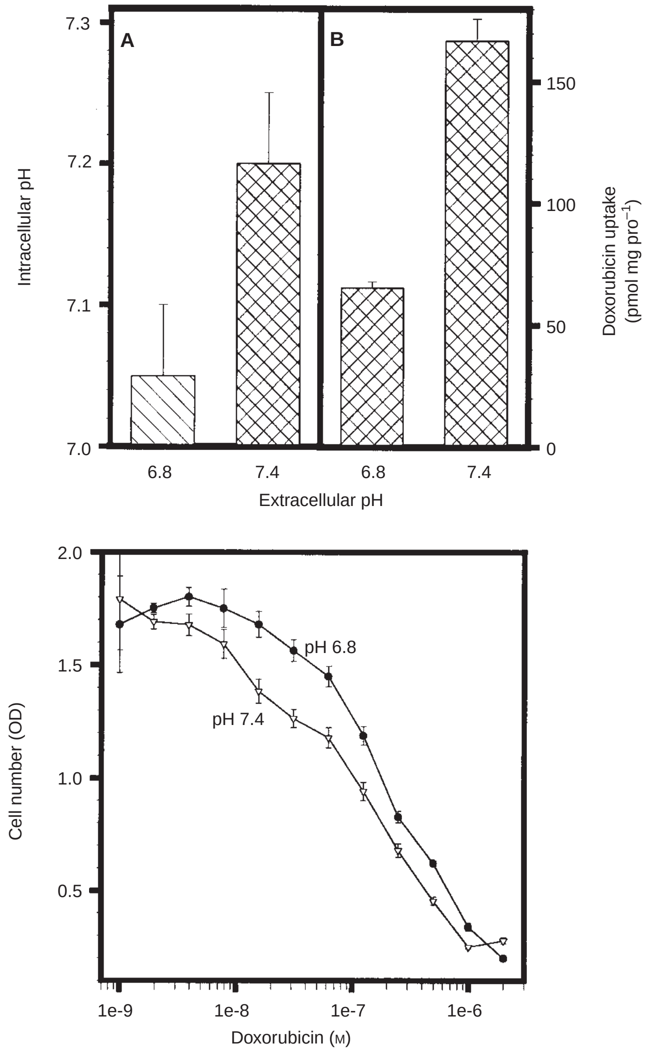

**Figure 1** In vitro effects of pH on doxorubicin chemotherapy. (**A**) Intracellular pH of MCF-7 cells in-vitro. MCF-7 cells grown on coverslips were loaded with SNARF-1 fluorescent dye and the intracellular pH was measured as described in Methods. (**B**) Doxorubicin levels in MCF-7 cells as a function of pH. MCF-7 cells were incubated with $^{14}$C doxorubicin for 30 min at medium pH of 6.8 or 7.4, after which time cells were washed and radioactivity determined (see Methods). Data are expressed as pmol mg protein$^{-1}$. (**C**) Cytotoxicity of doxorubicin as a function of pHe. MCF-7 cells were grown in 96-well plates to sub-confluence, at which time they were treated with doxorubicin at the indicated concentrations. Ninety-six hours later, media were removed and remaining cell numbers were determined by crystal violet staining, as described previously (Gillies et al, 1986). Data are shown for MCF-7 cells at pH 6.8 and 7.4. OD, optical density the cube root volumes from day 9 onward were fit for each mouse with non-zero tumour growth. Three-way analyses of variance were used to examine the effect of different experiments, injection (doxorubicin vs saline), and water (plain vs bicarbonate). The differences between experiments were not significant ($P$ = 0.767) and, hence, data from all experiments were pooled for further two-way analyses of variance. Tumour cell log kill was calculated from the equation $[(T–C)/3.32T_d]$, where T and C are the number of days it took for treated and control groups, respectively, to reach 1000 mm$^3$. $T_d$ is the exponential doubling time of the treated group (Corbett et al, 1982). T/C was calculated as the ratio of the median volumes of treated and control groups on day 20. T/C < 44% is considered significant by the Division of Cancer Treatment (National Cancer Institute), while a T/C value of < 10% (DN-2 level activity) is considered highly significant.

### RESULTS

### pH affects doxorubicin distribution and cytotoxicity in vitro

Solid tumours generally exhibit pHe values of 6.7–6.8 (Wike-Hooley et al, 1984; Griffiths, 1991; Gillies et al, 1994; McCoy et al, 1995; Raghunand et al, 1999*a*). In vitro, MCF-7 cells at medium pH values of 6.8 and 7.4 have pHi values of 7.05 ± 0.06 and 7.20 ± 0.07, respectively, as measured with fluorescence (Figure 1A). Thus, according to theory (see Methods), the pH gradient of +0.25 at pHe = 6.8 will yield an intracellular–extracellular doxorubicin ratio of 0.69 ± 0.07, whereas at pHe = 7.4, the ratio will be 1.36 ± 0.17 (Table 1). Hence, there will be a 1.97 ± 0.45-fold enhancement of the steady-state intracellular drug concentration as pHe is raised from 6.8 to 7.4. The empirical steady-state levels of $^{14}$C doxorubicin in MCF-7 cells at medium pH values of 6.8 and 7.4 show a 2.56 ± 0.25-fold enhancement (Figure 1B), which is in good agreement with theory.

The difference in intracellular levels of doxorubicin between low and high pHe values should give rise to differences in cytotoxicity. Figure 1C shows that, following a 24-h treatment, the $EC_{50}$ of doxorubicin in MCF-7 cells is 0.27 μm at pH 6.8, compared to 0.12 μm at pH 7.4. One-hour treatments with doxorubicin yielded essentially the same results, although both $EC_{50}$ values were higher. Similar data for doxorubicin have been reported elsewhere (Tannock and Rotin, 1989; Simon and Schindler, 1994). In the current study, the 2.25-fold enhancement of doxorubicin cytotoxicity at pH 7.4 compared to pH 6.8 is similar in magnitude to both theory and the empirical doxorubicin distribution ratio (cf. Table 1). Thus, the enhanced cytotoxicity at high pHe is likely due to increased partitioning of doxorubicin into cells.

### Bicarbonate raises pHe in vivo

While there is substantial evidence that doxorubicin is more effective at higher pHe, this phenomenon is of limited utility unless the pHe of tumours can be manipulated in vivo. For the current experiments, pHe was raised via sodium bicarbonate-induced metabolic alkalosis. However, the addition of bicarbonate could have important negative sequelae, since it is osmotically active and could cause hypernatraemia. To determine the maximum tolerated doses of bicarbonate, eight different cohorts of SCID mice were given $NaHCO_3$-supplemented water to drink ad libitum. Bicarbonate concentrations ranged from 0 to 250 mM. Animal weights and water consumption were monitored daily. For bicarbonate concentrations up to 225 mM, weight gain and subjective parameters (e.g. grooming behaviour and activity) were unaffected compared to water controls for up to 9 weeks (data not shown). In a separate experiment, animals were given 200 mM $NaHCO_3$ ad lib. Four to 10 days following commencement of bicarbonate therapy, pHi and pHe were measured using $^{31}$P MRS. A representative $^{31}$P spectrum from an untreated MCF-7 cell tumour is shown in Figure 2A. In this Figure, the pHe measured by 3-APP is 6.57 and the pHi measured by Pi is 6.95. Figure 2B shows the region containing the 3-APP resonance from a representative $^{31}$P MRS spectrum of

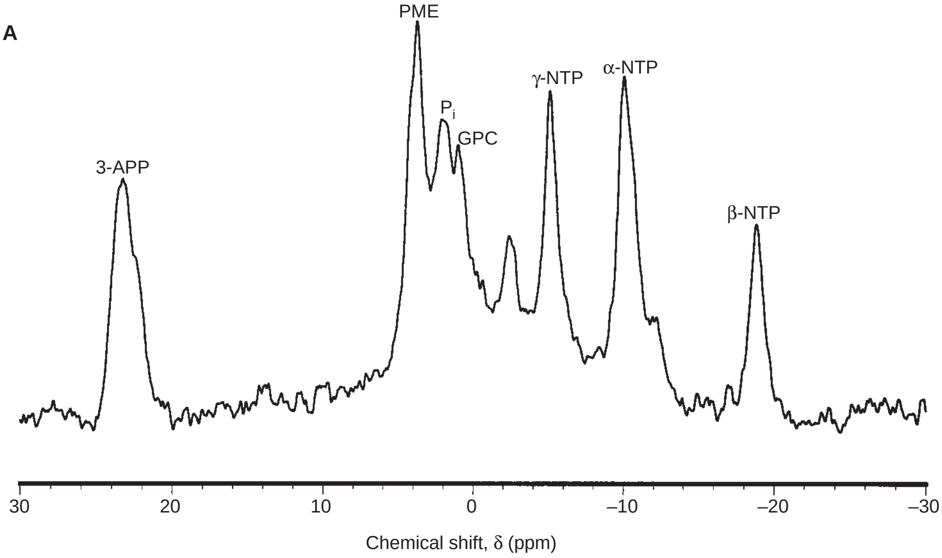

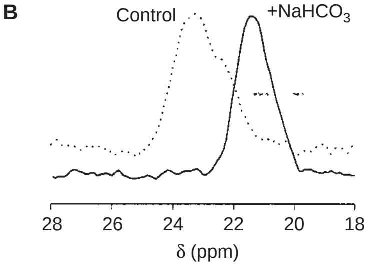

**Figure 2** Measurement of tumour pH with $^{31}P$ MRS. (**A**) In vivo $^{31}P$ NMR spectrum of MCF-7 tumour grown in mammary fat pad of severe combined immunodeficient (SCID) mouse. The in vivo $^{31}P$ spectra were processed by Fourier transformation with 15 Hz exponential line broadening and referenced to the α-resonance of NTP at –10.05 ppm. The intra and extracellular pH values were obtained from the chemical shifts of Pi and 3-APP respectively (McCoy et al, 1995; Raghunand et al, 1999*a*). Pi, inorganic phosphate; NTP, nucleoside triphosphates; PME, phosphomonoesters. (**B**) Downfield region of spectra from control and $NaHCO_3$-treated mice. Downfield region of $^{31}P$ MR spectra showing 3-APP resonance from MCF-7 tumours in SCID mice. The dotted line (downfield resonance) is from control mouse at pHe 6.57 and the solid line (upfield resonance) is from mouse given 200 mM $NaHCO_3$ ad libitum for 4 days, showing a pHe of 7.72. Acquisition conditions were identical to those in Figure 1A

MCF-7 tumours obtained 4 days following substitution of drinking water with 200 mM $NaHCO_3$. Note the shift in the position of the 3-APP resonance relative to controls indicating a significant alkalinization of pHe. All other resonances (Pi, PME, ATP) were statistically indistinguishable from the controls. In this example, the measured pHe was 7.72. Thus, $NaHCO_3$ can significantly increase pHe.

Results from all of the MRS-based pH measurements are shown in Figure 3A, expressed as a function of tumour size. Note that in untreated MCF-7 tumours (triangles), the pHe (open) and pHi (closed) decrease with approximately the same slope with increasing tumour size. Thus, the pH gradient is relatively constant at 0.22 ± 0.09 pH unit, consistent with previous reports (Raghunand et al, 1999*a*). In animals supplemented with $HCO_3^-$, the measured pHe values (open circles) are significantly higher than the measured pHi values (closed circles). This is significantly different than the pHe and pHi of control animals. The direction of the plasmalemmal (in–out) pH gradient is shown by arrows in the Figure and it is clear that the direction is reversed in all $NaHCO_3$-treated animals, compared to controls. The pHe reported by the 3-APP resonance in $NaHCO_3$-treated tumours is high, 7.84 ± 0.13 pH unit. The pHi values in the same animals were 7.39 ± 0.12. The imprecision in both the pHe and pHi measurements are likely higher due to these values being near the alkaline extreme of the titration curves for both 3-APP and Pi respectively. Nevertheless, the data clearly show that chronic bicarbonate therapy effectively alkalinizes the pHe in vivo, generating an (in–out) ΔpH of approximately – 0.45 pH unit, compared to a ΔpH of + 0.20 in untreated animals.

### Bicarbonate chemosensitizes tumour xenografts in vivo

According to the measured pH gradients, the theoretical cytosolic–extracellular drug ratio should be 1.6 in bicarbonate-treated animals, and 0.6 in controls, leading to a 2.5-fold increase in doxorubicin sensitivity (Table 1). To test this, MCF-7 tumours were grown in the mammary fat pads of 6-week-old female SCID mice, to sizes of 50–200 $mm^3$, and were forcibly randomized according to tumour size into four groups.

Groups A and C were given normal water ad libitum, while the water given to groups B and D was supplemented with 200 mM $NaHCO_3$. The start of the bicarbonate treatment was designated as day 1. On days 3, 7 and 11, animals in groups C and D were injected i.p. with doxorubicin while animals in groups A and B ($n$ = 10 each) received saline injections of the same volume, as per established protocols (Paine-Murrieta et al, 1997). The maximum tolerated dose (MTD) of SCID mice for doxorubicin under this regimen is a cumulative 6.7 mg $kg^{-1}$ (Paine-Murrieta et al, 1997). Initial experiments used three doses of doxorubicin at 2.0 mg $kg^{-1}$ (cohorts $C_1/D_1$) and this was reduced in later experiments to three doses at 1.6 mg $kg^{-1}$ (cohorts $C_2/D_2$) to reduce morbidity. In all

*© 1999 Cancer Research Campaign*

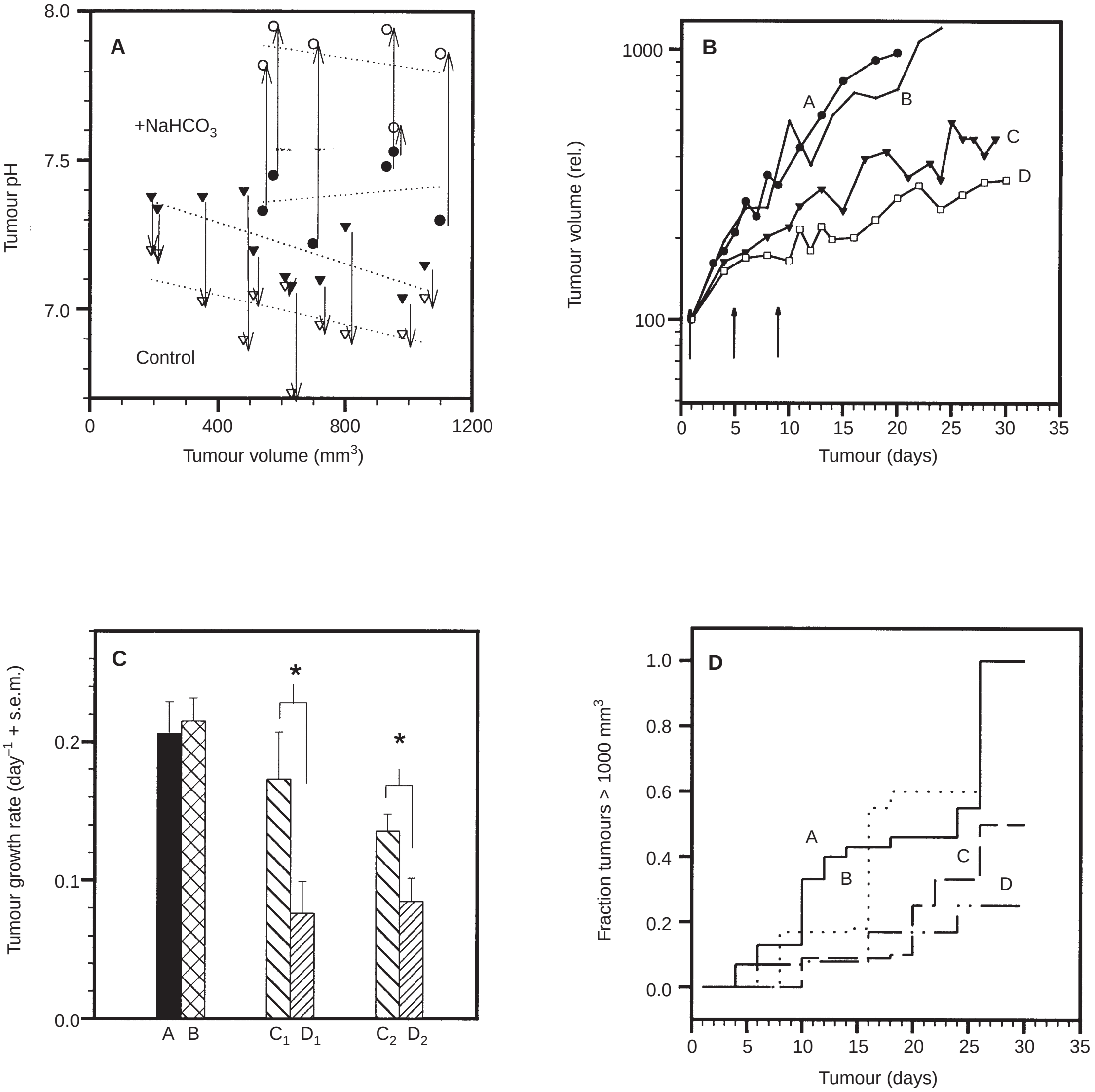

**Figure 3** In vivo effects of combination chemotherapy. (**A**) pHi and pHe of MCF-7 tumours in control (triangles) and bicarbonate-treated (circles) mice. pHi and pHe values were measured using $^{31}$P MRS, as described for Figure 1. These pHe data were collected 4–10 days following commencement of bicarbonate therapy. Closed symbols represent the intracellular pH values, measured with Pi, and the open symbols represent extracellular pH values, measured with 3-APP. Arrows indicate the direction of the intra- to extracellular gradient, and dotted lines represent independent linear regressions of the four data sets. (**B**) Tumour volumes as a function of time. MCF-7 tumours were grown in SCID mice to volumes of 50–200 mm$^3$, after which time they were randomized into four cohorts (A–D). Beginning on day 1, cohorts A and C received drinking water, whereas cohorts B and D received 200 mM $NaHCO_3$. On days 1, 5 and 9, animals of cohorts C and D were injected i.p. with doxorubicin (arrows), whereas animals from cohorts A and B were injected with saline. On day 15, animals of cohorts B and D were placed back on drinking water. For the purpose of display, tumour volumes were normalized to 100% on day 4. Data points represent the pooled means of 3–16 measurements at each time point from all experiments. (**C**) Specific growth rates. The rate of increase in tumour size was determined between days 9 and 15 for each animal by linear regression and expressed as fraction of surviving mice per day individual tumour. The illustrated data represent the mean ± standard deviation of these data sets binned by cohort. The cohorts were as described in Figure 3B, except that cohorts $C_1/D_1$ and $C_2/D_2$ were separated since they received doxorubicin at 2.0 and 1.6 mg kg$^{-1}$ respectively. *Indicates values were significantly different ($P < 0.03$) by Student's *t*-test. (**D**) Individual tumour growth curves. Percent of total tumours in a cohort greater than 1000 mm$^3$ are plotted as a function of time since beginning of experiments. Cohorts are as described for Figure 3B

experiments, the incidence of morbidity following drug treatment was identical between groups C and D. In parallel experiments, three doses of i.p. doxorubicin at 2.0 mg kg$^{-1}$ on days 1, 5 and 9 gave no evidence of vesication upon day 10 necropsy. Tumour volumes and animal weights were monitored every 2–3 days. For graphing purposes, tumour volumes for each mouse were normalized to day 1. As shown in Figure 3B, doxorubicin has a significant effect on the tumour growth rate, and this effect is greater in animals co-treated with bicarbonate. For statistical analyses, normalized data were used to calculate specific tumour growth rates for each tumour, from day 9 onward. The means of these normalized tumour growth rates are shown in Figure 3C. Regardless of dose, the tumour growth rate was significantly ($P \leq 0.03$) slower in the bicarbonate plus doxorubicin groups ($D_1$, $D_2$), compared to the doxorubicin alone groups ($C_1$, $C_2$) (Table 2). Furthermore, bicarbonate alone had no significant effect on the growth rate ($P > 0.4$, A vs B). Table 2 also shows that the tumour growth delays (T–C) were 15 and 22 days at 300 mm$^3$ for groups C and D respectively. However, since the slopes of the growth curves diverge, the T–C increased with time, and thus cannot be used to

*© 1999 Cancer Research Campaign* 

reliably calculate log cell kill. The T/C values calculated at day 20 were 0.47 (751/1571) and 0.29 (458/1571) for groups C and D respectively. A modified (Kaplan–Meier type) survival plot showing the fraction of remaining animals with small (< 1000 $mm^3$) tumours is shown in Figure 3D. These data show that animals receiving doxorubicin plus bicarbonate take significantly longer to develop larger tumours compared to the other groups.

### DISCUSSION

These data indicate that raising pHe increased the cytotoxic effectiveness of doxorubicin both in vitro and in vivo. In-vitro, raising the pHe from 6.8 to 7.4 resulted in a 2.25-fold enhancement of cytotoxicity and a 2.56-fold increase in intracellular doxorubicin concentrations. These data compared well to the 1.97-fold enhancement predicted from the calculated difference in doxorubicin distributions (Table 1). It is possible that the larger enhancement observed empirically, relative to theory, is due to sequestration of doxorubicin in acidic vesicles, which was not accounted for in the theoretical calculations (Altan et al, 1998; Raghunand et al, 1999*b*).

In vivo, in the SCID mouse–MCF7 tumour model system used here, the data clearly show that $NaHCO_3$ effectively raises the pHe for extended periods of time. Prolonged treatments with bicarbonate in drinking water did not have significant effects on animal well-being.

Nevertheless, for bicarbonate therapy to have clinical utility, the minimum required treatment time needs to be determined. Previous protocols involving bicarbonate treatment of humans have primarily been developed to counteract acidosis caused by exercise or renal failure (Passfall et al, 1997; Verbitsky et al, 1997). A problem in these protocols is poor patient compliance, with chronic bicarbonate ingestion. Clinical metabolic alkalinization with bicarbonate will be most successful if it is administered acutely, viz. shortly prior to the time of chemotherapy. Hence, experiments are underway to determine the time course of alkalinization and whether acute, intermittent alkalinization is as effective, compared to chronic bicarbonate administration.

Combination therapy with bicarbonate and doxorubicin resulted in a significantly lowered tumour growth rate compared to treatment with doxorubicin alone. In SCID mice, as in human patients, doxorubicin alone is not a highly effective cytotoxic agent with much heterogeneity of response. Many workers have used SCID mice to test doxorubicin chemotherapy (Liu et al, 1996; Cesano et al, 1997; Durand and LePard, 1997). However, a recent report indicates that doxorubicin is not effective to treat tumours in SCID mice, due to granulocyte toxicity to the host (Polin et al, 1997). This may be observed in our studies, since the tumour outgrowth did not occur with the same doubling time as the untreated tumours. Consequently, log kill values could not be reliably calculated. However, the T/C value measured at day 20 was 0.29 for group D, compared to 0.47 for group C. T/C < 44% is considered significant by the Division of Cancer Treatment (NCI). Thus, this measure indicates a meaningful boost in therapeutic effectiveness caused by raising the pH with bicarbonate. In other systems, such as sygeneic mice (16/C mammary adenocarcinoma in C3H mice), log kill from doxorubicin can be 2.5–3.0 (T Corbett, personal communication). Hence, experiments are planned using this more responsive mouse system, even though it is not a high connectivity model for human clinical response.

The growth rates resulting from the two different doxorubicin doses were not significantly different (i.e. $C_1$ vs $C_2$, $D_1$ vs $D_2$). However, the lower doxorubicin dose did reduce the mortality of the treated animals during the course of the experiments. At 2 mg $kg^{-1}$, half of the animals survived in both groups to day 30. At 1.6 mg $kg^{-1}$, only one animal from each group (out of eight) did not survive until day 30. It must be noted that doxorubicin-treated animals appeared more distressed than controls. By day 15, animals in groups C and D displayed poor grooming and urinary discharges. By day 30, all animals in group D were experiencing anal discharge as well. This suggests that, in addition to the tumours, bicarbonate also sensitized the intestinal epithelium to doxorubicin cytotoxicity. Since bicarbonate should only have this effect on tissues with low initial pHe, this might indicate that intestinal epithelial cells or colonic crypts have a naturally low pHe. This side-effect of combination bicarbonate–doxorubicin chemotherapy might be avoided with shorter bicarbonate treatment times, more selective alkalinizing agents, or protection of the intestinal epithelium with acid buffers or V-ATPase inhibitors. Despite the qualitatively poorer condition of group D animals, there was no significant difference in their body weights, compared to animals of group C.

Our calculations predict that alkalinization should provide a two- to threefold increase in the therapeutic efficacy of doxorubicin. This is significant, because many current clinical studies indicate that threefold accelerated dosing of doxorubicin is more effective in treating node-positive breast cancers (Bearman et al, 1997). However, doxorubicin has associated cardiotoxicity and there is a significant morbidity associated with accelerated dosing regimens. Thus, if two- to threefold increases in cytosolic doxorubicin could be achieved through manipulation of pH, it is likely that the therapeutic benefits could be realized without the increased morbidity associated with accelerated dosing.

In summary, the current data indicate that the pHe of human breast tumour xenografts is acidic and that this reduces the cytotoxicity of doxorubicin in vitro and in vivo. Tumour pHe can be chronically raised by treatment with sodium bicarbonate in the drinking water of tumour-bearing SCID mice, and this significantly increases the chemotherapeutic efficacy of doxorubicin chemotherapy in vivo.

### ACKNOWLEDGEMENTS

The authors would like to thank J-P Galons for helpful discussions, L Celaya for secretarial assistance, and TH Corbett, J Evelhoch and S Salmon for thoughtful evaluations of the manuscript. Support from the US Army Breast Cancer Initiative grants DAMD-17-94-J-4368 (RJG) and DAMD-17-96-1-6131 (ZMB) and NIH RO1-CA23074 is also gratefully acknowledged.

### REFERENCES

Altan N, Chen Y, Schindler M and Simon SM (1998) Defective acidification in human breast tumor cells and implications for chemotherapy. *J Exp Med* **187**: 1583–1598

Bearman SI, Overmoyer BA, Bolwell BJ, Taylor CW, Shpall EJ, Cagnoni PJ, Mechling BE, Ronk B, Baron AE, Purdy MH, Ross M and Jones RB (1997) High-dose chemotherapy with autologous peripheral blood progenitor cell support for primary breast cancer in patients with 4–9 involved axillary lymph nodes. *Bone Marrow Transplant* **20**: 931–937

Cesano A, Visonneau S, Rovera G and Santoli (1997) Synergistic effects of adriamycin and TALL-104 cell therapy against a human gastric carcinoma in vivo. *Anticancer Res* **17**: 1887–1892

© 1999 Cancer Research Campaign

Corbett TH, Roberts BJ, Trader MW, Laster WR Jr, Griswold DPJ and Schabel FM Jr. (1982) Response of transplantable tumors of mice to anthracenedione derivatives alone and in combination with clinically useful agents. *Cancer Treat Rep* **66**: 1187–1200
Durand RE and LePard NE (1997) Tumour blood flow influences combined radiation and doxorubicin treatments. *Radiother Oncol* **42**: 171–179
Gillies RJ and Deamer DW (1978) Intracellular pH: methods and applications. *Curr Top Bioenergetics* **9**: 63–87
Gillies RJ, Didier N and Denton M (1986) Determination of cell number in monolayer cultures. *Analyt Biochem* **159**: 109–113
Gillies RJ, Liu Z and Bhujwalla Z (1994) $^{31}$P-MRS measurements of extracellular pH of tumors using 3-aminopropylphosphonate. *Am J Physiol* 195–203
Griffiths JR (1991) Are cancer cells acidic? *Br J Cancer* **64**: 425–427
Liu C, Lambert JM, Teicher BA, Blattler WA and O'Connor R (1996) Cure of multidrug-resistant human B-cell lymphoma xenografts by combinations of anti-B4-blocked ricin and chemotherapeutic drugs. *Blood* **87**: 3892–3898
Martinez-Zaguilan R, Martinez GM, Lattanzio F and Gillies RJ (1991) Simultaneous measurement of intracellular pH and $Ca^{2+}$ using the fluorescence of SNARF-1 and Fura-2. *Am J Physiol* **260**: C297–C307
McCoy CL, Parkins CS, Chaplin DJ, Griffiths JR, Rodrigues LM and Stubbs M (1995) The effect of blood flow modification on ontra- and extracellular pH measured by 31P magnetic resonance spectroscopy in murine tumours. *Br J Cancer* **72**:
Negendank W (1992) Studies of human tumors by MRS: a review. *NMR Biomed* **5**: 303–324
Ordidge RJ, Bowley RM and McHale G (1988) A general approach to selection of multiple cubic volume elements using the ISIS technique. *Magn Reson Med* **8**: 323–331
Paine-Murrieta GD, Taylor CW, Curtis RA, Lopez MHA, Dorr RT, Johnson CS, Funk CY, Thompson F and Hersh EM (1997) Human tumor models in the severe combined immune deficient (*Scid*) mouse. *Cancer Chemother Pharmacol* **xx**: 1–6
Passfall J, Pai J, Spies KP, Haller H and Luft FC (1997) Effect of water and bicarbonate loading in patients with chronic renal failure. *Clin Nephrol* **47**: 92–98
Polin L, Valeriote F, White K, Panchapor C, Pugh S, Knight J, LoRusso, Hussain M, Liversidge E, Peltier N, Golakoti T, Patterson G, Moore and Corbett TH (1997) Treatment of human prostate tumors PC-3 and TSU-PR1 with standard and investigational agents in SCID mice. *Invest New Drugs* **15**: 99–108
Raghunand N, Altbach MI, Van Sluis R, Baggett B, Taylor CW, Bhujwalla ZM and Gillies RJ (1999*a*) Plasmalemmal pH-gradients in drug-sensitive and drug-resistant MCF-7 human breast carcinoma xenografts measured by 31P MR spectroscopy. *Biopharm Pharmacol* **57**: 309–312
Raghunand N, Martinez-Zaguilan R, Wright SH and Gillies RJ (1999*b*) pH and drug resistance II: turnover of acidic vesicles and resistance to weakly basic chemotherapeutic drugs. *Biochem Pharmacol* **57**: 1047–1058
Roos A (1978) Weak acids, weak bases, and intracellular pH. *Respir Physiol* **33**: 27–30
Simon SM and Schindler M (1994) Cell biological mechanisms of multidrug resistance in tumors. *Proc Natl Acad Sci USA* **91**: 3497–3504
Tannock IF and Rotin D (1989) Acid pH in tumors and its potential for therapeutic exploitation. *Cancer Res* **49**: 4373–4384
Taylor CW, Dalton WS, Parrish PR, Gleason MC, Bellamy WT, Thompson FH, Roe DJ and Trent JM (1991) Different mechanisms of decreased drug accumulation in doxorubicin and mitoxantrone resistant variants of the MCF7 human breast cancer cell line. *Br J Cancer* **63**: 923–929
Verbitsky O, Mizrahi J, Levin M and Isakov E (1997) Effect of ingested sodium bicarbonate on muscle force, fatigue, and recovery. *J Appl Physiol* **83**: 333–337
Warburg O (1956) On the origin of cancer cells. *Science* **123**: 309–314
Wike-Hooley JL, Haveman J and Reinhold HS (1984) The relevance of tumour pH to the treatment of malignant disease. *Radiother Oncol* **2**: 343–366

*© 1999 Cancer Research Campaign*
